# Habitual Constipation

**Published:** 1887-02

**Authors:** 


### HABITUAL CONSTIPATION.

This is one of the most common ailments that afflicts mankind; and one which very seriously affects the general health; and yet there is no disease in the whole catalogue that so often baffles the efforts of the physician to treat with success. Indeed few medical men thoroughly appreciate the seriousness of confirmed constipation, and too often it is tampered with, and really made worse, by the administration of purgatives, which give only temporary relief, leaving the bowels less disposed to move than before. It is conceded we believe, that purgatives, alone, will not cure constipation. The desideratum long sought—a remedy which will cure or greatly alleviate this distressing malady, seems to have been found in the Extract of Cascara Sagrada, and for several years, recently, the medical press has been teeming with testimonials as to its efficiency in constipation, espectally when due to atony of the muscular fibre. Our experience with this remedy dates back some six or eight years. We found Cascara Sagrada—the fluid extract prepared by Park, Davis & Co., to answer most admirably when many of the older remedies had failed. The house of Park, Davis & Co. deserve the thanks of the profession for having devised a menstuum for the exhibition of the drug, which had become recognized as nearly a specific in certain forms of constipation, one which is at once elegant and efficient. Cascara cordial is composed of a pure fluid extract of the drug, together with French brandy and aromatics; and when administered it seems not to disagree with the most delicate stomach. In our experience the cordial overcomes the torpidity of the bowels,not by irritation,of the terminal intestinal nerve filaments, as do purgatives, but by a *tonic effect* upon the muscular coats, whereby peristalsis is produced, and the bowel enabled to expel its contents. Proof of this is found in the fact that the evacuations are not thin and watery, and accompanied by griping, but, often, are of hard, solid consistency. There can be no doubt from our observation, that it stimulates and promotes the flow of bile also, and on that account, is an excellent "liver medicine," well adapted to those cases of constipation which are due to, or accompanied by, hepatic congestion. Under the daily use of Cascara Cordial alone, or in cases of mal assimilation, mixed with *malt* or maltine and pepsin—we have seen the appetite and digestion improve, the complexion clear up, and a sense of vigor imparted, which has not resulted from the use of any other drug.

We shall have more to say of this remedy another time. Meantime we call attention of our brethren who, perhaps like ourselves, have had trouble in treating confirmed constipation, to another valuable, and rational remedy.

Dr. A. V. Meigs, of Philadelphia, recommends the following combination of drugs, in pill form, for obstinate constipation.

℞ Ext. Belladonæ Gr. 1-12

" nucis vom: " ¼

" aloes " ⅛

" Rhei " ¼ in one pill, to be taken three times a day.

This is an excellent combination, and bye the bye, very similar to a small, well made, and perfectly soluble gelatine coated pill recently put on the market by Sharp & Dohme of Baltimore, under the name "Pil Lapacticæ." This formula is as follows:

℞ Aloin Gr. 1-4
Strychninæ " 1-60
Ext. Bellad " 1-8
Ipecac " 1-16 in one pill; dose one or two pills. There is no doubt that the addition of the minute dose of ipecac is an improvement, as Ringer & Murrell, and Fothergill testify to its value in very small doses, as an adjunct in such prescriptions.

